# HepParser: An Intelligent Software Program for Deciphering Low-Molecular-Weight Heparin Based on Mass Spectrometry

**DOI:** 10.3389/fchem.2021.723149

**Published:** 2021-09-09

**Authors:** Hui Wang, Yu Wang, Meijie Hou, Chunming Zhang, Yaojun Wang, Zhendong Guo, Dongbo Bu, Yan Li, Chuncui Huang, Shiwei Sun

**Affiliations:** ^1^ Key Lab of Intelligent Information Processing, State Key Lab of Computer Architecture, Big-data Academy, Institute of Computing Technology, Chinese Academy of Sciences, Beijing, China; ^2^ University of Chinese Academy of Sciences, Beijing, China; ^3^ Phil Rivers Technology, Beijing, China; ^4^ College of Information and Electrical Engineering, China Agricultural University, Beijing, China; ^5^ Institute of Biophysics, Chinese Academy of Sciences, Beijing, China

**Keywords:** LMWHs, mass spectrometry, glycosaminoglycans, isotopic distribution, computational method

## Abstract

Low-molecular-weight heparins (LMWHs) are considered to be the most successful carbohydrate-based drugs because of their wide use as anticoagulants in clinics. The efficacy of anticoagulants made by LMWHs mainly depends on the components and structures of LMWHs. Therefore, deciphering the components and identifying the structures of LMWHs are critical to developing high-efficiency anticoagulants. However, most LMWHs are mixtures of linear polysaccharides which are comprised of several disaccharide repeating units with high similarity, making it extremely challenging to separate and decipher each component in LMWHs. Here, we present a new algorithm named hepParser to decipher the main components of LMWHs automatically and precisely based on the liquid chromatography/mass spectrometry (LC/MS) data. When tested on the general LMWH using hepParser, profiling of the oligosaccharides with different degrees of polymerization (dp’s) was completed with high accuracy within 1 minute. When compared with the results of GlycReSoft on heparan sulfate samples, hepParser achieved more comprehensive and reasonable results automatically.

## Introduction

Heparin is a complex, linear polysaccharide, which belongs to the family of glycosaminoglycans (GAGs). Most heparins are comprised of ∼25 disaccharide repeating units of a glucuronic acid residue (GlcA) or iduronic acid residue (IdoA) 1,4 linked to a glucosamine residue (GlcN), with various substitution patterns of sulfation at the 2-O-position of the hexuronic acid residue (HexA), the 3-O-position, the 6-O-position, and/or the N-position of GlcN, and N-acetylation at GlcN (Linhardt, 2003; Wang and Chi, 2018). Low-molecular-weight heparins (LMWHs) are derived from heparin and possess similar primary structures. Compared to heparin, the average molecular weights of LMWHs are usually between ∼4,000 and ∼8,000 Da, containing ∼6–∼12 disaccharide units (Weitz, 1997; Li et al., 2012). The decrease of average molecular weight improves LMWHs’ bioavailability, including increasing *in vivo* half-life, enhancing pharmacology, changing activity profile, and reducing thrombin inhibitory activity. Owing to these improved properties, LMWHs have been widely used as clinical anticoagulants (Warkentin et al., 1995; Cohen et al., 1997; Linhardt and Gunay, 1999; Norrby, 2006; Bhaskar et al., 2012). Biological functions of LMWHs are closely related to specific components and structural diversity, and therefore, deciphering components and identifying structures are critical to developing high-efficiency anticoagulants (Hemkerl, 1992). However, it is unfeasible to dissociate each component in LMWHs completely. Besides complex components, derivatives with labile sulfate loss are greatly analogous to each other and are indistinguishable (Jones et al., 2011; Kailemia et al., 2012; Duan and Jonathan Amster, 2018). The complexity and high similarity of components put forward tremendous challenges for analyzing and sequencing LMWHs.

A variety of methods have been applied for parsing LMWHs such as liquid chromatography (LC), capillary electrophoresis (CE), and size exclusion chromatography (SEC). However, these techniques provide little precise structural information on LMWHs (Pervin et al., 1995; Mao et al., 2002; Guo et al., 2003). Proton and carbon nuclear magnetic resonance (NMR) spectroscopy can present the most detailed information of the primary structure of LMWHs (Li et al., 2012). However, large amounts of samples (e.g., hundreds of micrograms) are required for NMR analysis, and high-throughput analysis and detailed structural features cannot be achieved (Guerrini et al., 2007). Due to high sensitivity and rich structural information, mass spectrometry (MS), especially coupled to LC, has become the primary method in characterizing and sequencing LMWHs (Wolff et al., 2007; Kailemia et al., 2012; Kailemia et al., 2013). Nevertheless, peaks with multiple charges and noises due to experimental instruments or interfering impurity are usually present in mass spectra and significantly increase the difficulties in deciphering components of LMWHs.

Fortunately, some computational tools have been proposed to assist researchers in analyzing the components of LMWHs. GlycoWorkbench is a popular software program that can be used to interpret mass spectra, but automatic analysis of LMWHs’ components cannot be performed (Ceroni et al., 2008; Slysz et al., 2010). Maxwell et al. developed a software program called GlycReSoft to identify and quantify heparin components based on mass spectra deconvoluted by DeconTools (Slysz et al., 2010; Maxwell et al., 2012). The software may be of low efficiency for large LC/MS datasets due to the dependence on DeconTools for deconvolution (Mechref et al., 2013). Hu et al. developed an algorithm named HS-SEQ for *de novo* sequencing of heparan sulfate samples and assigning positions of acetate and sulfate groups on the oligosaccharide chains (Hu et al., 2014). However, HS-SEQ requires mass spectra with high resolution, and conflicts of assignment may cause incorrect identification (Duan and Jonathan Amster, 2018). Chiu et al. developed GAG-ID to sequence heparin mixtures by parsing LC-MS/MS data, whereas those mixtures require complete chemical derivatization to tackle labile sulfate modifications (Chiu et al., 2015).

In this work, we developed a new algorithm named hepParser to decipher main components of LMWHs based on the LC/MS data. HepParser eliminates the interferences of noisy isotopic peak clusters and spectra shifts on profiling results using the designed peak merging and peak calibration algorithms and conducts automatic analysis of LMWHs’ components with high speed and accuracy. Furthermore, with the assistance of a well-fitting model, theoretical isotopic distribution of a given mass can be produced by hepParser, and the unreliable isotopic peak clusters can be discarded based on the similarity between the experimental and theoretical isotopic distributions, which makes the profiling results more confident. HepParser has achieved an excellent performance on the tested general LMWH sample with 2–8 degrees of polymerization. We also compared the performance of hepParser with that of GlycReSoft on heparan sulfate samples. The results reported by hepParser were more comprehensive and reasonable.

## Materials and Methods

### Experimental Methods

Heparin sodium (0.1%, 125 U/ml) extracted from porcine intestinal mucosa was obtained from Bioroyee (Beijing, China), and LMWHs were prepared through degradation using sodium nitrite and heparinase I. Sodium nitrite (2.95% of the Heparin) dissolved in 2.5 mol/l hydrochloric acid was added to heparin, which was then allowed to react at 35°C for 90 min. Sodium hydroxide was added to the solution to terminate the degradation (pH, 10), and sodium borohydride (1% of heparin) was injected to the solution for reduction overnight. Excess acetic acid was added to the above solution (pH, 4.0) and allowed to react for 15 min. The redundant sodium borohydride was therefore eliminated, and then 2 mol/L sodium hydroxide was added to neutralize the solution. Methanol was added to the reacted solution (67%), and the solution was stored at 4°C for 24 h. The solution was centrifuged, and the precipitates were primary LMWHs. The LMWHs were finally obtained after lyophilization using a 1,000 Da dialysis bag. Exhaustive digestion of the prepared LMWHs with heparinase I was performed at 25°C for 48 h. Briefly, 100 μl 0.2 IU/ml heparinase I dissolved in 10 mM monobasic potassium phosphate (pH = 7) was added to 50 μg LMWHs in the presence of 100 μl sodium/calcium acetate solution (pH = 7, containing 2 mM calcium acetate and 0.1 mg/ml bovine serum albumin). After incubation, the reacted solution was heated at 100°C for 2 min to inactivate the enzymes and further filtered on a 0.22 μm filter prior to LC/MS analysis.

LC/MS data were acquired on LMWH samples using Acquity Xevo G2-S Q-TOF UPLC/MS systems (Waters, Milford, MA). An Acquity UPLC BEH C18 column (2.1 mm × 100 mm, 1.7 μm particles) was used for chromatographic separations. The column temperature was maintained at 40 C throughout the separation, and a flow rate of 0.5 ml/min was used. 10 μl of the 0.2 mM digested LMWHs dissolved in water was injected for each separation. A binary solvent system was used for gradient elution. Solvent A was composed of 5% acetonitrile in water, and solvent B consisted of 80% acetonitrile in water. Both mobile phases contained 15 mM of pentylamine or hexylamine (PTA or HXA, ion-paring reagents) and 50 mM 1,1,1,3,3,3-hexafluoro-2-propanol (HFIP, buffering agent). MS analysis was performed on a Waters Xevo G2-S quadrupole time-of-flight (Q-TOF) mass spectrometer equipped with an electrospray ionization (ESI) source. All the MS spectra were obtained in negative mode, and the mass range was 0–1,200 Da with a scanning rate of 0.5 s. The source temperature and the desolvation temperature were 120°C and 200°C.

### Computational Methods

**FIGURE 1:**
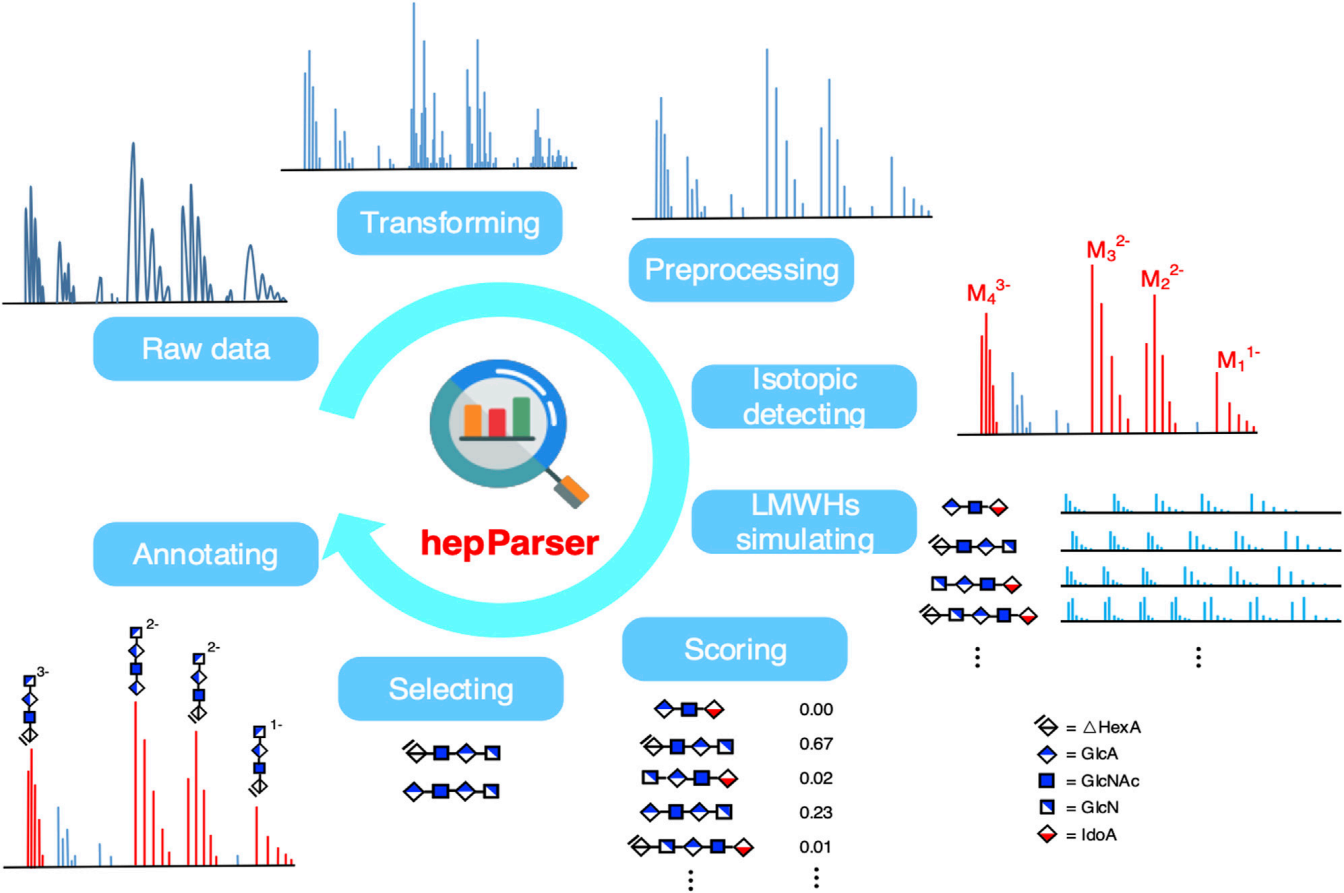
Workflow of hepParser to decipher components of LMWHs.

HepParser aims to uncover the components of LMWHs based on the MS spectra of the sample of interest. The pipeline of hepParser software is shown in Figure 1. In summary, hepParser would firstly perform three preprocessing steps for the given spectrum, followed by detecting the isotopic peak clusters and determining the charge of each cluster. Then, all possible components of LMWHs in a reasonable m/z range would be enumerated according to the composition rules of LMWH structures. Subsequently, the proposed score function would give a matching score for each component. At last, hepParser would greedily select components with high scores and annotate them on the original spectrum.

#### Data Preprocessing

To make the downstream LMWHs’ profiling more precise and efficient, three preprocessing steps were carried out for the selected mass spectrum from total ion chromatograms (TICs), including peak merging, peak denoising, and peak calibration.

a) Peak Merging

In order to read and process the original MS data more conveniently, we first converted them to the “mzML” format by MSConvert (Chambers et al., 2012) with the default setting. However, this conversion may split intensive peaks into several low peaks, which would lead to the shift of peak center and the increase of meaningless matching.

To fix the over-segmentation problem, we divided all peaks into several groups according to the trend of intensity change of adjacent peaks. The intensity of peaks in each group first increased and then decreased when all peaks were sorted by m/z. Then, all peaks in the same group were merged as a new peak, the m/z value is the centroid of all these peaks’ m/z, and the intensity is the sum of these peaks’ intensity.

**FIGURE 2:**
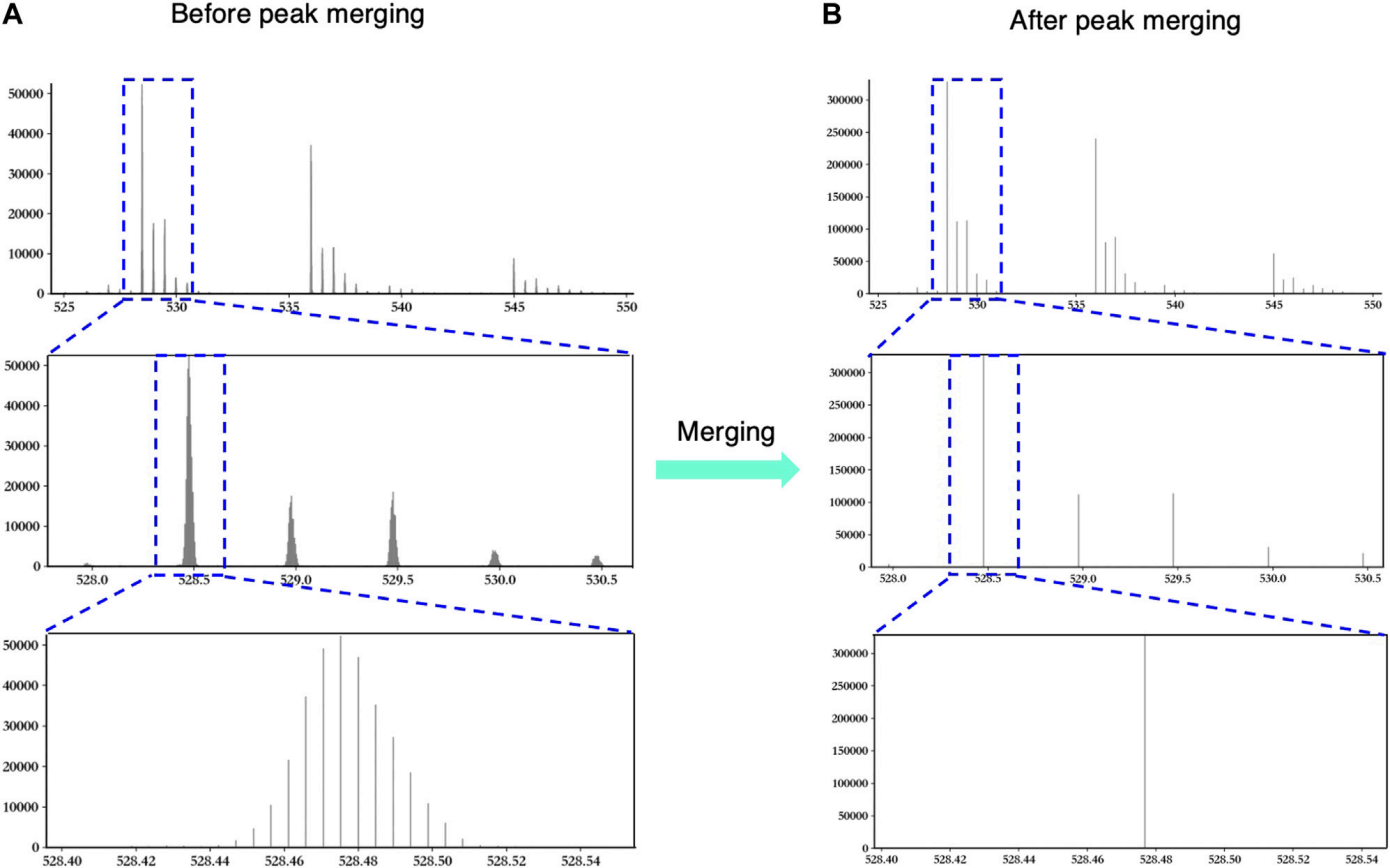
An example to explain the necessity and validity of peak merging. The left three windows show the original peaks before peak merging **(A)**, and the right three windows show the merged peaks after peak merging **(B)**. The blue dashed boxes represent the regions that are zoomed in.

Figure 2 shows an example to explain the necessity and validity of peak merging vividly. In Figure 2A, the original MS data show the over-segmentation when we keep zooming in the peak. Obviously, those peaks in the third window in Figure 2A should be merged as one peak. As shown in Figure 2B, hepParser solved the over-segmentation problem after peak merging.

b) Peak Denoising

MS spectra usually contain a large quantity of noisy peaks which would interfere in the profiling results greatly. Using hepParser, two denoising methods were applied to exclude noises including instrumental noises. If a low intensity value is observed for several times (default 1,000) in one spectrum, all peaks with this intensity will be treated as the instrumental noise. Therefore, all these peaks will be filtered out. In addition, a peak will also be considered the noise when its absolutive intensity is lower than the given threshold (500 in this study) or its relative intensity is lower than 0.001 (the relative intensity of the highest peaks was set as 1).

c) Peak Calibration

To correct the possible spectra’s shift caused by a mass spectrometer and data preprocessing, five peaks with the highest intensity in this spectrum are selected to pre-match with all LMWHs’ components. Differences in the exact relative molecular weight (MW) of each matched component and the peak’s m/z are recorded. Then, the optimal shift value which minimizes the sum of all m/z differences is calculated and calibrated for the given spectrum.

#### Isotopic Peak Cluster Detection

The detection of isotopic peak clusters and the determination of charge state are of great significance for ESI-MS spectra analysis and have a direct influence on the accuracy of the subsequent matching process. HepParser first detected all possible isotopic clusters as the candidates and then estimated their possibilities by the similarity between candidates’ intensity distributions and theoretical distributions.

a) Isotopic Cluster Candidate Extraction

Firstly, three parameters should be set before extraction, including the max possible charge state (default 5), the max peak number considered for each cluster (default 5), and the tolerance of peak matching (default 20 ppm). Then for each possible charge state, hepParser will search each peak in the spectrum to determine whether it is an isotopic peak cluster candidate by checking the differences in m/z between the peak and the surrounding peaks. If more than three peaks (including itself) satisfy the give tolerance, these peaks will be considered an isotopic peak cluster with the corresponding charge state. Afterward, hepParser extracted all possible isotopic peak clusters and recorded their charge states and intensity distributions.

b) Isotopic Peak Cluster Filtering

In order to obtain more reliable isotopic peak clusters, an intensity fitting model which can calculate the theoretical intensity distribution at the given m/z was trained to discard the cluster with an unreasonable intensity distribution. Firstly, numerous theoretical LWMHs’ components whose relative molecular masses are distributed in a wide range were enumerated. Then, the theoretical isotopic cluster intensity distributions of possible charge states were estimated using the Brain algorithm (Dittwald et al., 2013). Afterward, we fitted the first to last isotopic peak intensity in every isotopic cluster by the fourth-degree polynomial fitting algorithm, respectively. After calculating the theoretical cluster intensity distribution for each isotopic cluster candidate, the Jensen–Shannon divergence between the theoretical distribution and the experimental distribution was utilized to measure their qualities, and candidates with more than 0.9 similarity score (range 0–1) will be considered reliable isotopic peak clusters.

#### Low-Molecular-Weight Heparins’ Theoretical Spectra Simulation

A comprehensive component database which contains all possible components of LMWHs was constructed by enumerating components satisfying the composition rules of LMWH structures. Each component is recorded as a tuple with seven elements, and the representation of each element is as follows: [*△HexA, HexA, GlcN, Ac, SO3, Levoglucosan, Anhydromannitol*]. The simulated theoretical MS^1^ spectra of each component contain all possible peaks with different charge states and isotopic peaks. In addition, considering that LMWH components may lose chemical groups (such as sulfate groups) during mass spectrometry experiments, we also simulate all possible derivatization peaks and corresponding isotopic peak clusters in theoretical MS^1^ spectra of each component. The possible lost groups include $SO_{3},NH,NHSO_{3},\;\text{and }COO$. The user can control the maximum number of lost groups in one component (default 2).

#### Scoring

Each component is scored according to the matching of theoretical isotopic peak clusters and reliable experimental isotopic peak clusters. The score function is as follows:

$$S_{c}=\underset{p_{e},p_{t}}{\sum }\beta \ln\left(I_{p_{e}}+1\right)\left(1-JS\left(p_{e},p_{t}\right)\right)$$, (1)

where $p_{e}$ and $p_{t}$ are the matched experimental and theoretical isotopic peak clusters and $I_{p_{e}}$ is the total relative intensity of $p_{e}$. The logarithmic $I_{p_{e}}$ is aimed at measuring the effect of peak intensity for this score. $JS\left(p_{e},p_{t}\right)$ represents the Jensen–Shannon divergence of $p_{e}$ distribution and $p_{t}$ distribution, which shows the similarity of $p_{e}$ and $p_{t}$ distributions. β is the derived weight coefficient to control the effect of derived isotopic peaks, which is equal to 0.9 for derived $p_{t}$ or otherwise equal to 1. Obviously, the more the number of matched isotopic clusters, the more intensive the matched isotopic cluster, and the more similar the matched isotopic cluster, the higher the score for that component.

#### Component Selection

After scoring all enumerated LMWHs’ components, hepParser will report several of them as the final results to annotate the original spectrum. The component selection should guarantee the following two constraints:

i. Most isotopic peaks in the original mass spectrum can be explained by the selected components.
ii. Each component selected needs to make sufficient and irreplaceable contributions to the interpretation of the original mass spectrum.

To determine the optimal selection for the given spectrum, a global score function which controls the number of selections and a next candidate searching function are designed as follows to guide hepParser:

$$S_{g}=\alpha^{n}\overset{n}{\underset{i=1}{\sum }}\underset{p_{e},p_{t}\in E_{i}-H}{\sum }\beta \ln\left(I_{p_{e}}+1\right)\left(1-JS\left(p_{e},p_{t}\right)\right),$$ (2)

$$C_{next}=\underset{i\in unselected}{\arg\max}\left\{\underset{p_{e},p_{t}\in E_{i}-H}{\sum }\beta \ln\left(I_{p_{e}}+1\right)\left(1-JS\left(p_{e},p_{t}\right)\right)\right\},$$ (3)

where $S_{g}$ represents the score of the current selection and n represents the number of candidates which have been selected. α is the penalty factor to control the candidates’ number in the final selection (default 0.99). The specific score (noted as $S_{sp}$) for each i is the variant of $S_{C}$ in Eq. 2, where $E_{i}$ represents all the matched experimental isotopic peak clusters of the $i^{\text{th}}$ candidate and H represents all experimental isotopic peak clusters that have been explained by the first i−1 candidates. Therefore, only those isotopic peak clusters that cannot be matched by the first i−1 candidates will be calculated for the $i^{\text{th}}$ candidate. Meanwhile, $C_{next}$ will greedily choose the candidate with the highest $S_{sp}$ as the next one to be considered. Finally, the selection with the highest $S_{g}$ will be considered the optimal component collection for the given spectrum.

#### Significance Testing

To further measure the reliability of the reported components, a test of significance was performed after component scoring and selection. The null hypothesis (H_0_) is that the component is not in the sample, which means that all matching peaks of the component are accidental matches. Now, we need to calculate the probability of the above event’s occurrence and obtain its *p*-value.

Firstly, we should calculate the probability (denoted as p_m_) that one theoretical peak of the component is randomly matched by the experimental spectrum:

$$p_{m}=\frac{\sum_{i=1}^{n}tolerance\;of\;Tp_{i}}{total\;m/z\;range\;of\;E}$$ (4)

where “total m/z range of E”represents the distance from the leftmost peak to the rightmost peak of all peaks in the experimental spectrum, n represents the number of theoretical peaks of the component in the range of experimental spectra, and “$tolerance\;of\;Tp_{i}$” represents the size of the tolerance window of the *i* ^th^ theoretical peak, which can be calculated by its m/z and ppm.

Suppose that the number of theoretical peaks which are randomly matched (denoted as X) follows a binomial distribution $B\left(n,p_{m}\right)$, the probability density function is as follows:

$$P\left(X=k\right)=\left(\begin{array}{c} n\\ k \end{array}\right)p_{m}^{k}{\left(1-p_{m}\right)}^{n-k}.$$ (5)

Then, for each component with k matched peaks, the *p*-value can be calculated by

$$pvalue=F\left(X\geq k\right)=\overset{n}{\underset{i=k}{\sum }}P\left(X=i\right).$$ (6)

Because the hypothesis test with multiple comparisons may give a false-positive result, we applied the “Benjamini and Hochberg” (BH) method (Benjamini and Hochberg, 1995), one of the most commonly used methods to control the false discovery rate (FDR), to adjust the obtained *p*-values. At last, we used -log10 fold change to transform the adjusted *p*-values (denoted as $-log_{10}p_{adj}$). According to our experience, the component with $-log_{10}p_{adj}$>3 is reliable.

#### Annotation

The last step for hepParser is annotating the spectrum by the components selected in step 5. Every matched isotopic peak in the experimental spectrum will be labeled by the corresponding component with the charge state and the loss derivatization information.

#### Open-Source Public Archive

HepParser is developed using Python 3.7, and the source code, sample data, and tutorial are available at the following GitHub website: https://github.com/Sunmile/hepParser.

## Results and Discussion

### Performance on Low-Molecular-Weight Heparin Sample

The capability of deciphering components of LMWHs with high confidence from mass spectra was the primary goal of the development of hepParser. To this aim, a series of test experiments were carried out on the LMWH sample, including profiling of HPLC fractions with dp2 and dp4.

**FIGURE 3:**
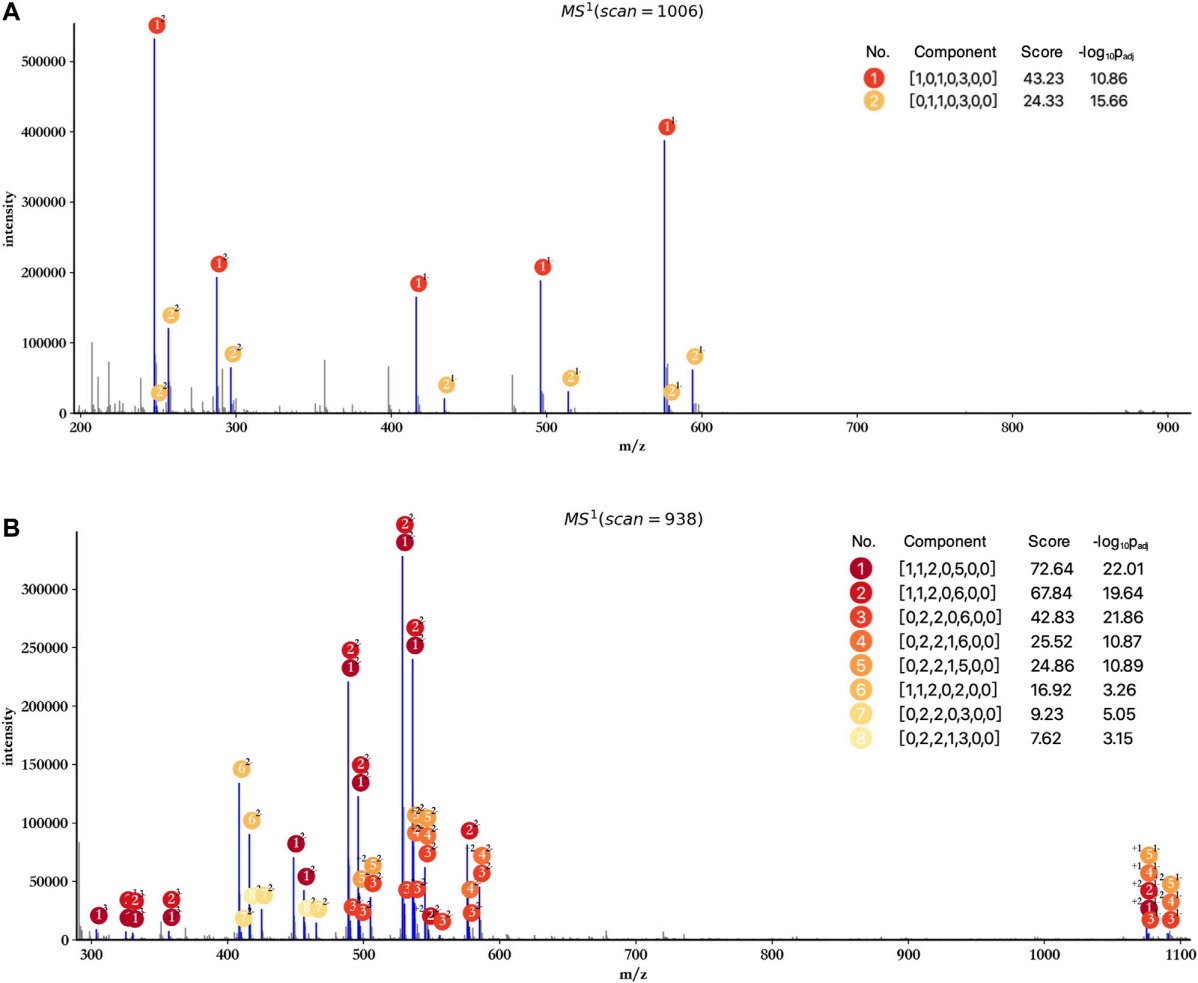
Profiling results of dp2 **(A)** and dp4 **(B)** oligosaccharides. The scan number represents the sampling point order in the mass spectrometry experiment. Each matched isotopic peak cluster in experimental spectra is marked in blue and annotated by the number and charge state of corresponding components. The score of each component is calculated by Eq.1, which indicates the number and quality of matching peaks for the component.

The profiling results of dp2 and dp4 oligosaccharides are illustrated in Figures 3A,B. HepParser deciphered two main components in dp2 oligosaccharides which can explain most isotopic peak clusters with the consideration of losing different derivatizations and different charge states. Meanwhile, eight main components were determined in dp4 oligosaccharides and also can interpret most isotopic peak clusters, indicating the high accuracy of hepParser. In addition, less than 1 minute was required for hepParser to obtain the profiling results, which manifests the overwhelming advantage of hepParser in analysis speed compared to the manual analysis.

HepParser can export the profiling results to an editable table for downstream analysis. The exported details in the table include experimental matched m/z values, charge states, the orders of matched isotopic peaks in the clusters, the compositions of parsed LMWHs’ components, and the lost groups of each matched component. Taking dp4 oligosaccharides, for instance, the exported details are listed and shown in Table 1.

**TABLE 1:** The exported detailed profiling result of dp4 oligosaccharides.

m/z	Charge	Isotopic peak	Components	Loss
303.6865	3	1	[1, 1, 2, 0, 5, 0, 0]	[2, 0, 0, 0]
325.3366	3	1	[1, 1, 2, 0, 5, 0, 0]	[0, 0, 1, 0]
325.3366	3	1	[1, 1, 2, 0, 6, 0, 0]	[1, 0, 1, 0]
330.3379	3	1	[1, 1, 2, 0, 5, 0, 0]	[1, 0, 0, 0]
330.3379	3	1	[1, 1, 2, 0, 6, 0, 0]	[2, 0, 0, 0]
356.9920	3	1	[1, 1, 2, 0, 5, 0, 0]	[0, 0, 0, 0]
356.9920	3	1	[1, 1, 2, 0, 6, 0, 0]	[1, 0, 0, 0]
408.5484	2	1	[1, 1, 2, 0, 2, 0, 0]	[0, 1, 0, 0]
410.0510	2	1	[0, 2, 2, 0, 3, 0, 0]	[0, 1, 1, 0]
416.0533	2	1	[1, 1, 2, 0, 2, 0, 0]	[0, 0, 0, 0]
416.5541	2	1	[0, 2, 2, 1, 3, 0, 0]	[0, 0, 1, 1]
425.0584	2	1	[0, 2, 2, 0, 3, 0, 0]	[1, 0, 0, 0]
448.5271	2	1	[1, 1, 2, 0, 5, 0, 0]	[1, 0, 1, 0]
456.0327	2	1	[1, 1, 2, 0, 5, 0, 0]	[2, 0, 0, 0]
456.5334	2	1	[0, 2, 2, 1, 3, 0, 0]	[0, 1, 0, 1]
465.0354	2	1	[0, 2, 2, 0, 3, 0, 0]	[0, 0, 0, 0]
488.5050	2	1	[1, 1, 2, 0, 5, 0, 0]	[0, 0, 1, 0]
488.5050	2	1	[1, 1, 2, 0, 6, 0, 0]	[1, 0, 1, 0]
490.0054	2	1	[0, 2, 2, 0, 6, 0, 0]	[0, 0, 2, 0]
496.0106	2	1	[1, 1, 2, 0, 5, 0, 0]	[1, 0, 0, 0]
496.0106	2	1	[1, 1, 2, 0, 6, 0, 0]	[2, 0, 0, 0]
496.5116	2	1	[0, 2, 2, 1, 5, 0, 0]	[0, 0, 1, 1]
497.5096	2	1	[0, 2, 2, 0, 6, 0, 0]	[1, 0, 1, 0]
505.0155	2	1	[0, 2, 2, 0, 6, 0, 0]	[2, 0, 0, 0]
505.0155	2	3	[0, 2, 2, 1, 5, 0, 0]	[1, 0, 0, 1]
528.4828	2	1	[1, 1, 2, 0, 5, 0, 0]	[0, 1, 0, 0]
528.4828	2	1	[1, 1, 2, 0, 6, 0, 0]	[0, 0, 1, 0]
529.9821	2	1	[0, 2, 2, 0, 6, 0, 0]	[0, 1, 1, 0]
535.9886	2	1	[1, 1, 2, 0, 5, 0, 0]	[0, 0, 0, 0]
535.9886	2	1	[1, 1, 2, 0, 6, 0, 0]	[1, 0, 0, 0]
536.4898	2	1	[0, 2, 2, 1, 5, 0, 0]	[0, 1, 0, 1]
536.4898	2	1	[0, 2, 2, 1, 6, 0, 0]	[0, 0, 1, 1]
537.4889	2	1	[0, 2, 2, 0, 6, 0, 0]	[0, 0, 1, 0]
544.9936	2	1	[0, 2, 2, 0, 6, 0, 0]	[1, 0, 0, 0]
544.9936	2	3	[0, 2, 2, 1, 5, 0, 0]	[0, 0, 0, 1]
544.9936	2	3	[0, 2, 2, 1, 6, 0, 0]	[1, 0, 0, 1]
547.4698	2	3	[1, 1, 2, 0, 6, 0, 0]	[0, 1, 0, 1]
555.9730	2	2	[0, 2, 2, 0, 6, 0, 0]	[0, 1, 0, 1]
575.9665	2	1	[1, 1, 2, 0, 6, 0, 0]	[0, 0, 0, 0]
576.4674	2	1	[0, 2, 2, 1, 6, 0, 0]	[0, 1, 0, 1]
577.4669	2	1	[0, 2, 2, 0, 6, 0, 0]	[0, 1, 0, 0]
584.9724	2	1	[0, 2, 2, 0, 6, 0, 0]	[0, 0, 0, 0]
584.9724	2	3	[0, 2, 2, 1, 6, 0, 0]	[0, 0, 0, 1]
1074.992	1	3	[1, 1, 2, 0, 5, 0, 0]	[0, 0, 0, 0]
1074.992	1	2	[0, 2, 2, 1, 5, 0, 0]	[0, 1, 0, 1]
1074.992	1	3	[1, 1, 2, 0, 6, 0, 0]	[1, 0, 0, 0]
1074.992	1	2	[0, 2, 2, 1, 6, 0, 0]	[0, 0, 1, 1]
1075.993	1	1	[0, 2, 2, 0, 6, 0, 0]	[0, 0, 1, 0]
1090.999	1	1	[0, 2, 2, 0, 6, 0, 0]	[1, 0, 0, 0]
1090.999	1	3	[0, 2, 2, 1, 5, 0, 0]	[0, 0, 0, 1]
1090.999	1	3	[0, 2, 2, 1, 6, 0, 0]	[1, 0, 0, 1]

### The Fitting Curves of Theoretical Isotopic Distribution

**FIGURE 4:**
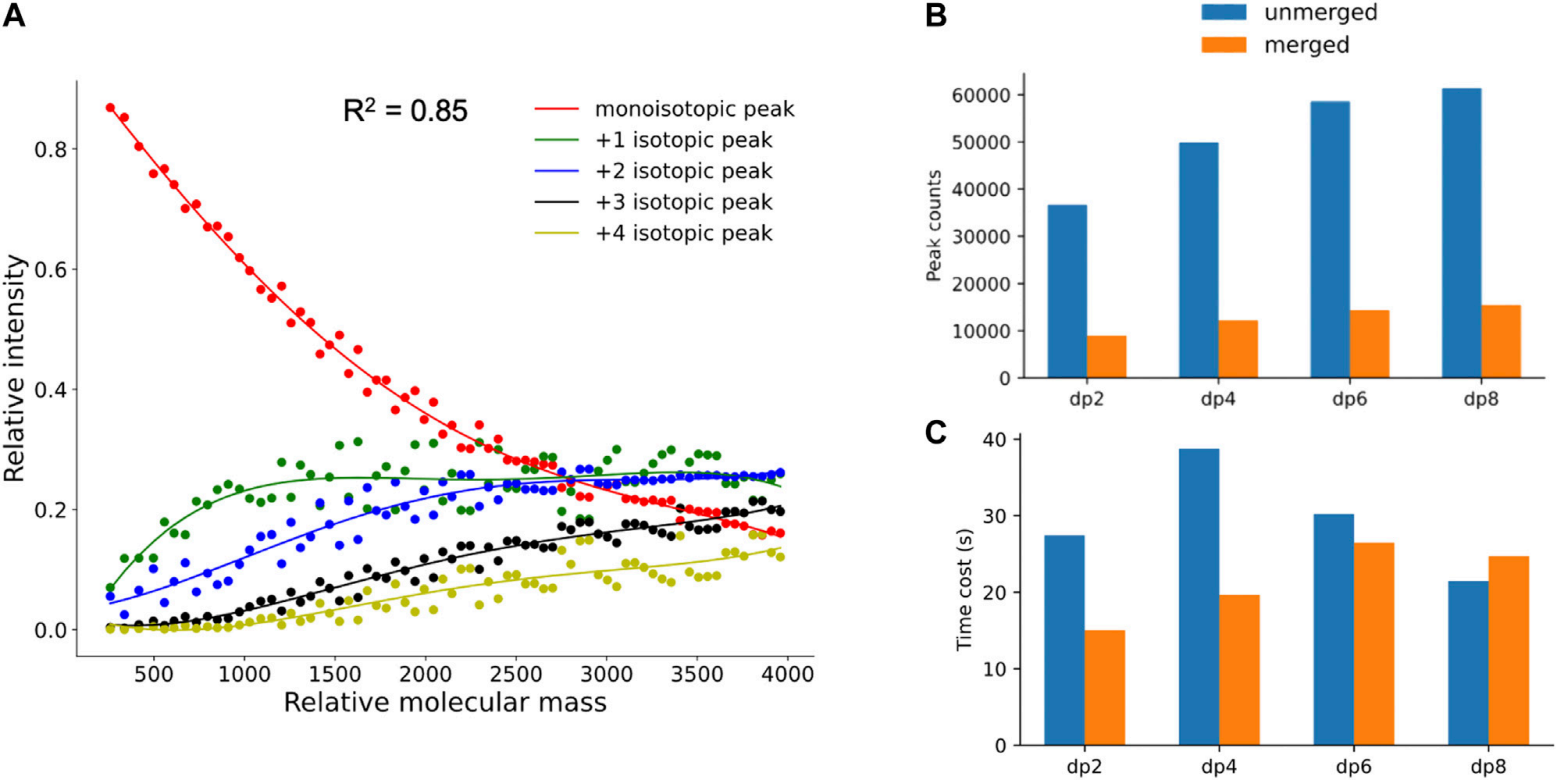
**(A)** Fitting results of theoretical isotopic peak distribution. **(B,C)** Comparison of peak number and time cost between unmerged spectra and merged spectra in dp2, dp4, dp6, and dp8 oligosaccharides.

As mentioned in *Materials and Methods*, to filter out the unreliable isotopic peak clusters, hepParser would train a fitting model with the polynomial fitting algorithm to calculate the theoretical isotopic distribution of a given mass. 70 compositions with different masses which are evenly distributed in the range of 200–4,000 Da were sampled from 6,167 possible compositions to calculate the theoretical isotopic distribution for fitting. As shown in Figure 4A, the fourth-degree polynomial fitting algorithm has a good performance in this fitting. The average goodness of fit (*R* ^2^) is 0.85 for the five fitting curves, which indicates that the fitting model has the ability of simulating theoretical isotopic distributions to estimate the confidence of each isotopic peak cluster candidate.

### Validity for Peak Merging and Peak Calibration

To verify the necessity and validity of peak merging and peak calibration in data preprocessing, the comparisons between spectra with and without the peak merging step were conducted. As shown in Figure 4B, the peak numbers in spectra of oligosaccharides with different degrees of polymerization were all greatly reduced after peak merging. As illustrated in Figure 4C, less time was required for hepParser to decipher dp2, dp4, and dp6 oligosaccharides from the MS spectra after peak merging. However, more time was required to decipher dp8 oligosaccharides from the MS spectra after peak merging. The main reason is that the relative amount of dp8 fraction was small, and multiple peaks with low intensity were produced in the MS spectra. It is likely that most isotopic peaks were filtered out in the MS spectra of dp8 oligosaccharides without peak merging.

**FIGURE 5:**
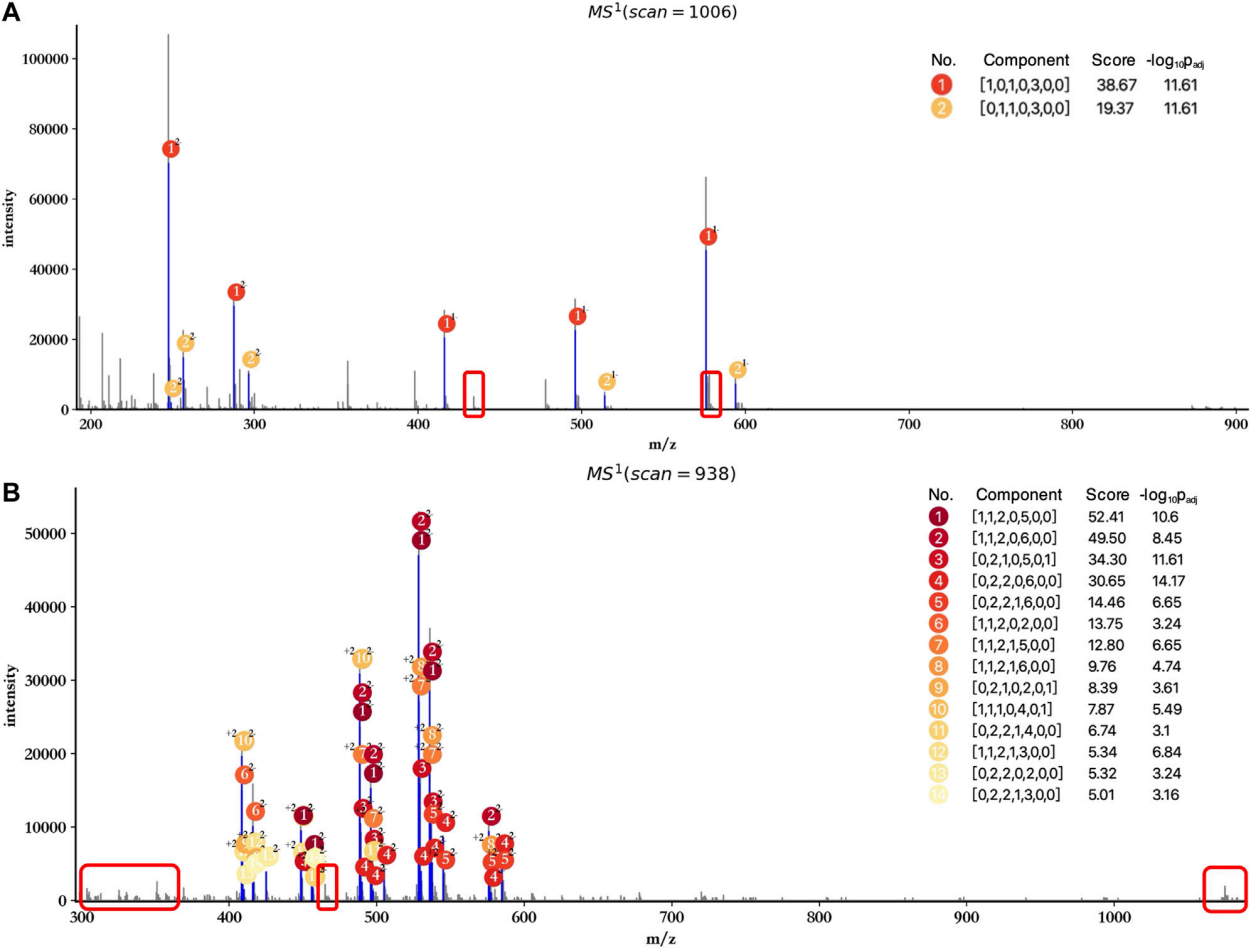
Profiling results of dp2 **(A)** and dp4 **(B)** oligosaccharides without peak merging and peak calibration. The regions marked by red rectangles contain isotopic peak clusters with low intensity which can be interpreted in Figure 3.

In addition, attention has to be paid to the profiling performance without peak merging and peak calibration. As shown in Figure 5, the score and significance of most matched components decreased significantly compared to the results obtained after peak merging and peak calibration (Figure 3). Few monoisotopic peaks can be assigned to oligosaccharide components, and isotopic peak clusters with low intensity cannot be interpreted by any components (marked by red rectangles) when merging and calibration were not performed. Therefore, peak merging and peak calibration are crucial for LMWHs’ profiling using hepParser.

### Annotation With a Single Component

**FIGURE 6:**
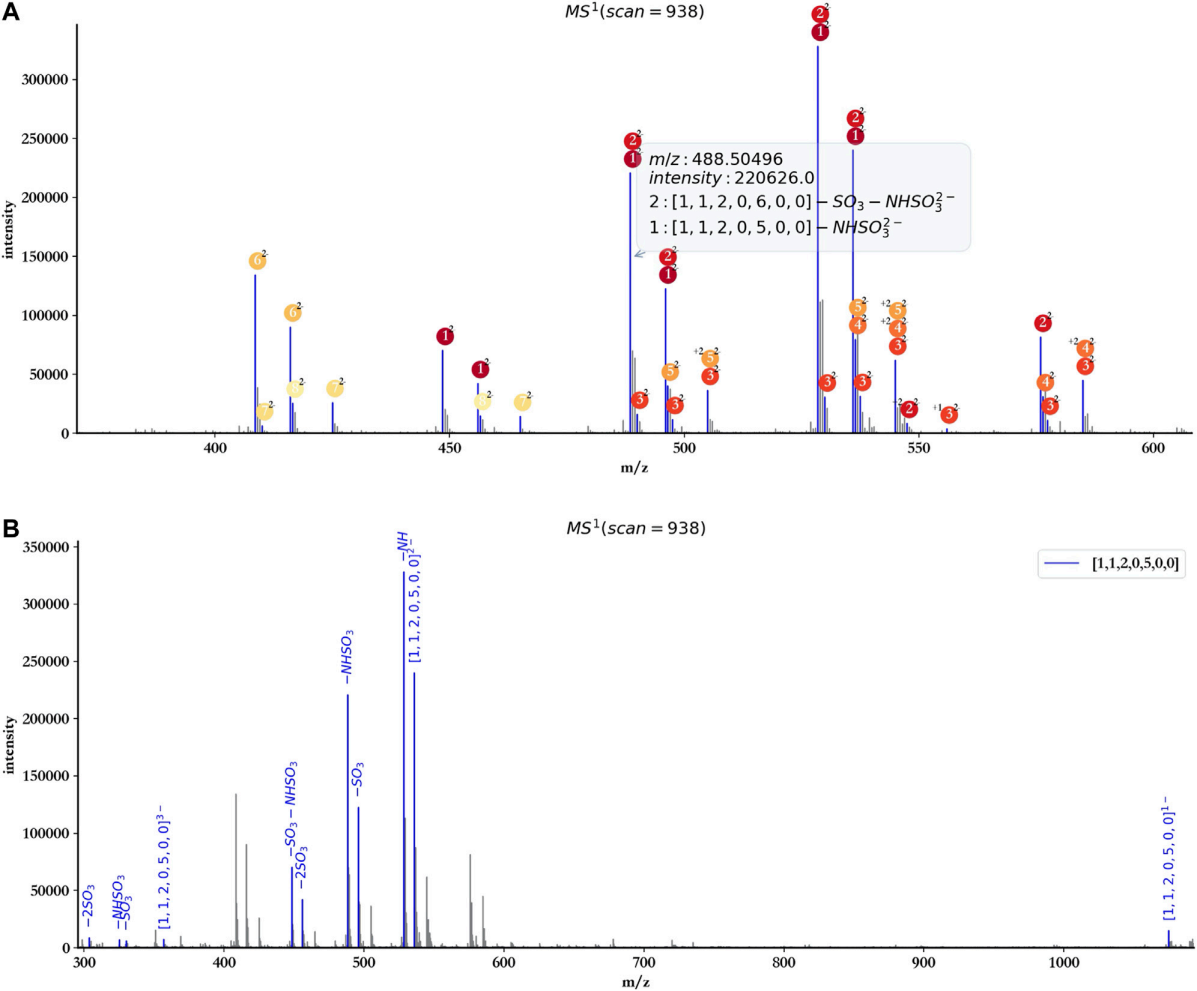
Tips window example **(A)** and annotation with a single component ([1, 1, 2, 0, 5, 0, 0]) of dp4 oligosaccharides **(B)**.

To facilitate the user to check the detailed information of each annotated peak, hepParser would show a tips window when the user hovered the cursor over the peak (Figure 6A).

The user also can annotate the spectrum with a single component using hepParser. As shown in Figure 6B, using the component [1, 1, 2, 0, 5, 0, 0] to annotate the spectrum of dp4 oligosaccharides, hepParser can label the matched isotopic peaks more clearly, including the information of possible lost groups, which will be useful for the downstream analysis.

### Comparison With GlycReSoft

To further validate the effectiveness, we compared the results reported by hepParser with those provided by GlycReSoft on the triplicate HS sample. The version of GlycReSoft we used is 1.0, which is available at https://code.google.com/archive/p/glycresoft/downloads.

**FIGURE 7:**
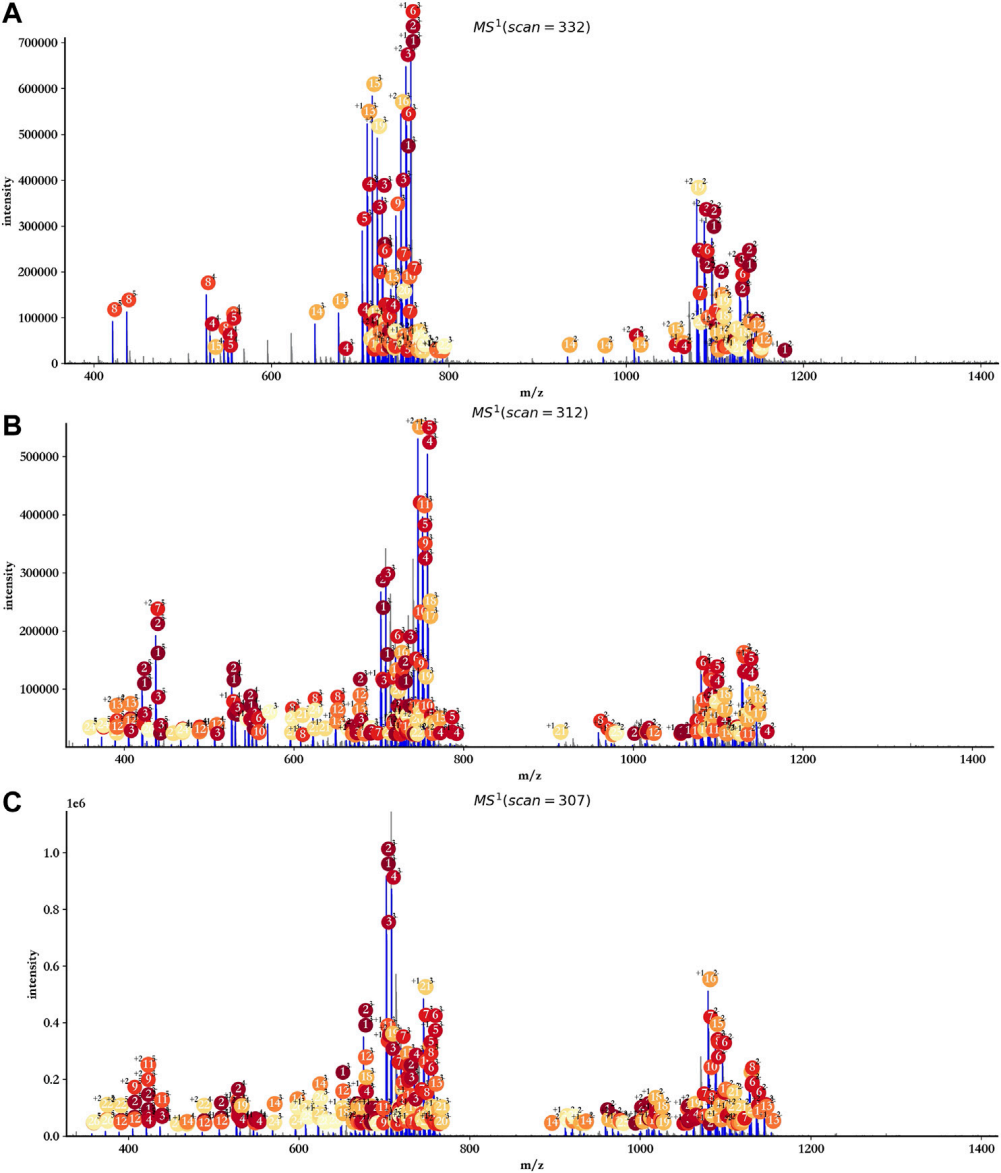
Annotation results of triplicate HS samples using hepParser. **(A)** hn042408-01.mzML. **(B)** hn042408-03.mzML. **(C)** hn042408-06.mzML.

As the data provided by GlycReSoft were mainly from dp8 oligosaccharides of heparan sulfate, oligosaccharides with degree of polymerization from 7 to 9 were mainly considered by hepParser with the setting of mass accuracy at 20 ppm, and Levoglucosan and Anhydromannitol residues were ruled out. The main annotation results are shown in Figure 7. Almost all reliable isotopic peak clusters were interpreted by hepParser very well. The detailed matching results and reporting components can be found in Supplementary Tables S1–S3.

**FIGURE 8:**
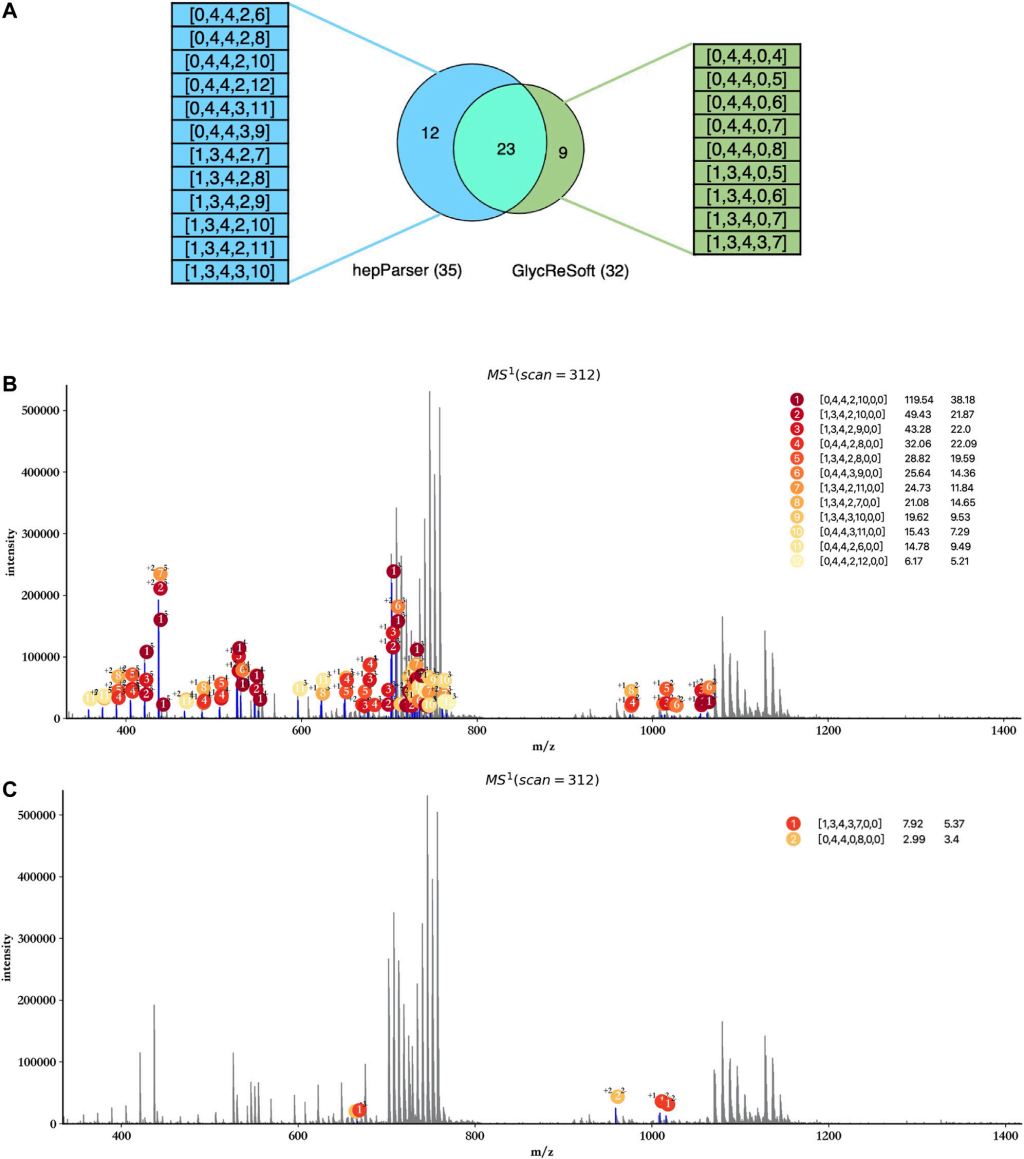
Comparison between hepParser and GlycReSoft results of triplicate HS samples. **(A)** Relationship of hepParser and GlycReSoft results. The 12 distinct components of hepParser are on the left (in blue), while the nine distinct components of GlycReSoft are on the right (in green). **(B)** Annotation results using the 12 distinct components of hepParser. **(C)** Annotation results using the nine distinct components of GlycReSoft (other seven components which matched no peak are not shown).

In view of the fact that GlycReSoft gives the component results of dp8 in the public sample data, we focused on the comparison of the dp8 oligosaccharides reported by hepParser and GlycReSoft. Before the comparison, we first transformed the results of hepParser to the format of GlycReSoft results. What we need to declare here is that one component of hepParser may be transformed to several components of GlycReSoft for the consideration of two possible groups lost in hepParser. For instance, [0, 4, 4, 0, 12, 0, 0] would be transformed to [0, 4, 4, 0, 12], [0, 4, 4, 0, 11], and [0, 4, 4, 0, 10]. As shown in Figure 8A, hepParser deciphered 35 dp8 oligosaccharides, while GlycReSoft reported 32, in which 25 components were shared by them. The distinct components of hepParser and GlycReSoft are extracted and shown on the left (12 of hepParser) and the right (9 of GlycReSoft) in Figure 8A. The detailed components in this comparison can be found in Supplementary Table S4.

Then, we compare the ability of interpreting the experimental spectrum of two groups’ distinct components and show it in Figures 8B,C. The 12 distinct components of hepParser can interpret amounts of peaks including some intensive peaks, and all components obtained high matching scores and high significance (Figure 8B), which indicated that they did exist in the sample data and the results of hepParser were reasonable. In contrast, there was no sufficient evidence to prove the existence of the nine distinct components of GlycReSoft. Only two of them can interpret five low intensity peaks (Figure 8C), which indicated the accuracy of hepParser to a certain extent.

## Conclusion

An open-source software program named hepParser was developed and applied for profiling of LMWHs. The efficiency was significantly improved through the peak merging strategy, and components of LMWHs were automatically analyzed with high accuracy. The general LMWH was analyzed in this proof-of-concept study, and profiling of oligosaccharides with different degrees of polymerization was successfully performed with high speed. As known to us all, deciphering components of LMWHs based on LC/MS data often involves time-consuming manual efforts and professional prior knowledge. The developed hepParser in this study can deal with the produced data rapidly and provide components of LMWHs automatically, which should facilitate analysis and functional studies of LMWHs. Structure identification of main components of LMWHs based on multistage mass spectrometry is planned for the future.

## Data Availability

The original contributions presented in the study are included in the article/Supplementary Material, and further inquiries can be directed to the corresponding authors.
